# STAT3 SH2 Domain Aspartic Acid 661 Mutations Activate Immune Gene Programs

**DOI:** 10.1111/jcmm.71015

**Published:** 2026-01-13

**Authors:** Hye Kyung Lee, Gyuhyeok Cho, Jichun Chen, Aaron B. Schultz, Sung‐Gwon Lee, Chengyu Liu, Priscilla A. Furth, Neal S. Young, Jungwook Kim, Alejandro Villarino, Lothar Hennighausen

**Affiliations:** ^1^ Section of Genetics and Physiology, National Institute of Diabetes and Digestive and Kidney Diseases, US National Institutes of Health Bethesda Maryland USA; ^2^ Department of Chemistry Gwangju Institute of Science and Technology (GIST) Gwangju Korea; ^3^ Hematology Branch, National Heart, Lung, and Blood Institute, National Institutes of Health Bethesda Maryland USA; ^4^ Department of Microbiology and Immunology Miller School of Medicine, University of Miami Miami Florida USA; ^5^ Sylvester Comprehensive Cancer Center, University of Miami Miami Florida USA; ^6^ Transgenic Core, National Heart, Lung, and Blood Institute, US National Institutes of Health Bethesda Maryland USA

**Keywords:** D661 variants, gain‐of‐function, hematologic malignancies, immune dysregulation, STAT3

## Abstract

The conserved aspartic acid residue D661 within the STAT3 SH2 domain is a recurrent mutational hotspot in hematologic malignancies, including T‐cell large granular lymphocytic leukaemia, myelodysplastic syndromes and acute lymphoblastic leukaemia. To define the functional consequences of distinct STAT3^D661^ variants, we integrated computational, structural and in vitro and in vivo genetic approaches. AlphaMissense and PolyPhen‐2 classified all four STAT3^D661^ variants (D661Y, D661V, D661N and D661H) as pathogenic. ClinVar classified D661Y and D661V as variants of uncertain significance. AlphaFold 3‐based modelling predicted that D661Y and D661V strongly promoted SH2‐TAD‐mediated dimerization, while D661N and D661H exerted weaker structural effects. Functional in vitro assays in *Stat3*‐deficient T cells demonstrated a gain‐of‐function (GOF) hierarchy of the STAT3 variants (D661Y ≈ V > H > *N*) resulting in activation of canonical STAT3 target genes and immune transcriptional programs. In vivo, only STAT3^D661H^ mice were viable, displaying reduced CD4^+^ T cells, expansion of memory CD8^+^ T cells and enhanced immune gene expression. Collectively, our findings define a gradient of STAT3 D661 GOF variants, consistent with in vitro and in vivo experiments. D661Y and D661V mutants exhibited stronger transcriptional activity in T cells with impaired viability of mice carrying these variants.

## 1 Introduction

Signal transducer and activator of transcription (STAT) proteins are central transcription factors that mediate cytokine and growth factor signalling, orchestrating diverse processes in immune regulation, haematopoiesis and oncogenesis [1]. Within the STAT family, STAT3 is predominantly activated by a range of cytokines, including IL‐6, IL‐10, IL‐21 and IL‐23 [2, 3] and recognises the canonical gamma‐activated sequence (GAS) motif (TTCnnnGAA) [4], reflecting its critical roles in T cell proliferation, NK cell function and growth regulation [5, 6]. Phenotypically, STAT3‐deficient mice exhibit impaired Th17 differentiation, defective humoral immunity and increased susceptibility to infection, partly due to loss of IL‐21 induction and impaired RORγt and IL‐23R expression—key STAT3‐dependent signals essential for Th17 lineage commitment and function [7, 8, 9]. Functioning within the Janus kinase (JAK)–STAT pathway, STAT3 becomes activated upon phosphorylation by JAKs, enabling dimerization via its Src Homology 2 (SH2) domain, nuclear translocation and transcriptional regulation of target genes. Dysregulation of this pathway—often through somatic variants in JAK2 or STAT3—can result in gain‐ or loss‐of‐function effects that disrupt immune homeostasis and promote malignant transformation [10, 11, 12].

STAT3 is activated downstream of a wide spectrum of cytokines, chemokines, growth factors and GPCR‐coupled signals. In addition to the classical IL‐6 family (IL‐6, IL‐11, IL‐27, OSM, LIF, CNTF) [13, 14, 15], activation is driven by IL‐10 family cytokines (IL‐10, IL‐19, IL‐20, IL‐22, IL‐24, IL‐26) [16, 17, 18], Th17‐associated cytokines (IL‐21, IL‐23) [15] and select γc‐cytokines such as IL‐7 and IL‐9 [16, 19]. Haematopoietic regulators including G‐CSF, GM‐CSF, M‐CSF, SCF, TPO and type I interferons also signal through STAT3. Growth‐factor RTKs (EGF/TGF‐α/epiregulin, PDGF, VEGF, FGF, HGF) [17, 19] and GPCR‐linked pathways such as sphingosine‐1‐phosphate, angiotensin, thrombin and inflammatory chemokines further contribute to STAT3 activation [13, 16]. While over fifty STAT3‐activating ligands have been described, those highlighted here represent the most relevant to immune regulation—the primary context of this study.

Germline STAT3 mutations fall into two mechanistically distinct categories. Loss‐of‐function (LOF) variants, typically in the DNA‐binding or SH2 domain, reduce STAT3 phosphorylation and transcriptional activity, causing autosomal‐dominant Hyper‐IgE syndrome with eczema, recurrent infections, high IgE and impaired Th17 development [20, 21, 22, 23]. In contrast, gain‐of‐function variants (GOF), commonly in the SH2 domain, enhance STAT3 dimerization and signalling, resulting in immune dysregulation, autoimmunity and lymphoproliferation. The D661 substitutions studied here represent SH2‐domain GOF mutations and are biologically and clinically distinct from LOF variants associated with Hyper‐IgE syndrome [24, 25, 26, 27, 28, 29, 30].

A mutational hotspot within STAT3 lies at the conserved aspartic acid residue D661 in the SH2 domain, which is critical for normal dimerization and activity [31]. Missense substitutions at this site (D661Y, D661V, D661H, D661N) have been reported in T‐cell large granular lymphocytic (T‐LGL) leukaemia, anaplastic large cell lymphoma (ALCL), myelodysplastic syndromes (MDS), acute lymphoblastic leukaemia (ALL) and other hematologic disorders [31, 32, 33, 34]. Beyond oncogenic roles, germline STAT3 variants are associated with immune disorders such as hyper‐IgE syndrome and autoimmune cytopenias [24, 25], underscoring its dual contribution of STAT3 to both malignancy and immune regulation. Among D661 variants, D661V and D661Y appear to promote constitutive STAT3 activation by enabling cytokine‐independent dimerization and nuclear localization, leading to persistent transcriptional activity, clonal lymphocyte expansion and immune dysregulation [31, 32, 33, 34]. Clinically, D661 mutations have been detected in both T and NK cell compartments [33, 35, 36] and have been linked to phenotypes such as increased mean corpuscular volume (MCV) [37], altered cytokine profiles and disrupted balance between pro‐ and anti‐inflammatory responses [38, 39, 40]—a reflection of STAT3's central role in regulating immune homeostasis, lymphocyte function and inflammatory signalling, which underlies its contribution to both autoimmunity and tumorigenesis [11, 41]. Despite these associations, the precise mechanistic consequences of D661 variants remain incompletely defined.

In this study, we investigated the functional consequences of four distinct STAT3^D661^ variants, 661Y, 661V, 661H and 661N. Through in silico and in vitro modelling, molecular analyses and the introduction of these human variants into the mouse genome, we gained insight into their impact on STAT3 signalling and immune cell pathobiology. We also integrated findings from the STAT5B^Y665F^ mutation to examine how the structurally related STAT3 and STAT5 transcription factors function both independently and cooperatively in regulating immune responses and cytokine signalling networks. Together, these insights provide a mechanistic framework for understanding STAT3–STAT5 crosstalk and highlight STAT3^D661^ as a potential biomarker and therapeutic target in leukaemia and immune‐mediated disorders.

## 2 Materials and Methods

### 2.1 Animals

All animals were housed and handled according to the Guide for the Care and Use of Laboratory Animals (8th edition) and all animal experiments were approved by the Animal Care and Use Committee (ACUC) of National Institute of Diabetes and Digestive and Kidney Diseases (NIDDK, MD) and the University Animal Care and Use Committee (University of Miami), and performed under the NIDDK animal protocol K089‐LGP‐23.

Stat3 floxed mice were generated as described [42] and backcrossed to C57BL/6J background for more than 10 generations. These mice were subsequently crossed with *Cd4*‐Cre mice (strain: 022071 from Jackson Labs, USA) to generate Stat3^flox/flox^ *Cd4*‐Cre^+/−^ mice, which lack STAT3 selectively in T cells. Wild‐type controls were *Cd4*‐Cre^−/−^ littermates or C57BL/6J mice were purchased from Jackson Labs (Strain: 000664). Both male and female mice were used, and all experimental cohorts were sex‐ and age‐matched.

CRISPR/Cas9 and base editing targeted mice were generated using C57BL/6N mice (Charles River) by the Transgenic Core of the National Heart, Lung and Blood Institute (NHLBI). Single‐guide RNAs (sgRNA) were obtained using Thermo Fisher Scientific's In Vitro Transcription Service (Table S5). Single‐strand oligonucleotide donor was obtained from IDT (Table S5). A single‐strand oligonucleotide donor contained the desired D (GAT) to Y/V/H (TAT, GTC, CAT, respectively) change. For the STAT3^D661^ mutant mice, D661Y/V/H sgRNA (20 ng/μL) was first mixed with Cas9 protein (IDT) to form Cas9 RNP complex, which was co‐microinjected with the oligo template into zygotes collected from superovulated C57BL/6N female mice (Charles River Laboratories) using a Nepa21 electroporator (Nepa Gene Co) following procedures described by Kenako [43]. The microinjected or electroporated zygotes were cultured overnight in M16 medium (Millipore Sigma) at 37°C with 6% CO_2_. Those embryos that reached 2‐cells stage of development were implanted into the oviducts of pseudo pregnant surrogate mothers (Swiss Webster mice from Charles River), anaesthetised with Ketamine (100 mg/kg) and Xylazine (10 mg/kg). All mice born to the foster mothers were genotyped by PCR amplification and Sanger sequencing (Quintara Biosciences) with genomic DNA from mouse tails. Mice aged 2–11 months were used in experiments. Mice aged 3 months were used for experiments examining immune cell function. Tissues were collected from 3‐month‐old male mice following euthanasia by CO_2_ inhalation (40% chambers' volume per minute) and were either used immediately or stored at −80°C.

### 2.2 STAT3 Variant Constructs

Mouse *Stat3* cDNA was synthesised based on reference sequence for the most abundant mRNA isoform (ENSMUST00000127638.8), then cloned into an MoMLV‐based plasmid vector (MigR1) immediately upstream of a dual internal ribosome entry sequence (IRES) and GFP cassette (synthesis and cloning by Genscript). STAT3 variants were generated by site directed mutagenesis of control STAT3 based on COSMIC annotations: D661H = c.1981G>C, D661N = c.1981G>A, D661V = c.1982A>T, D661Y = c.1981G>T (mutagenesis by Genscript). For retroviral packaging, STAT3 plasmids and pCL‐Eco ‘helper’ plasmid were co‐transfected into 293 T cells (ATCC) using Lipofectamine (Invitrogen), then virus‐containing supernatants collected 48 h later.

### 2.3 Isolation of *Stat3* Deficient T Cells and In Vitro Stimulation

The *Cd4‐Cre* transgene was introduced into *Stat3* floxed mice resulting in the deletion of the *Stat3* locus in both CD4 and CD8 cells at the ‘double positive’ stage of thymic development. Starting population are sorted naive CD4+ T cells (live, CD4 + CD44 lowCD25‐neg) from poled lymph nodes and spleens. The sequenced population are sorted RV‐transduced CD4+ T cells (live, CD4 + GFP+). Cells were transduced with an empty retroviral vector or vectors encoding the native and mutant STAT3 isoforms cultured for 48 h with IL‐2 and subsequently subjected to FACS analysis and RNA‐seq. T cells are activated with anti‐TCR and anti‐CD28 antibodies prior to retroviral transduction. The isoform of STAT5 that was packaged into retrovirus was Stat3‐203 (ENST00000404395.3), classified by EMSEMBL as ‘canonical’. This was then used as a template for the site‐directed mutagenesis to produce the Stat3 variants.

### 2.4 Cell Purification, Culture and Transduction

Mesenteric (mLN) and peripheral lymph nodes (pLN; inguinal, brachial, axillary and superficial cervical) were dissected from 8 to 16‐week‐old mice and processed to single‐cell suspensions by mechanical dissociation through 70 μM cell strainers. Suspensions were then normalised to 1 × 10^6^ cells/mL and stimulated with plate bound anti‐CD3ε (10 μg/mL; clone: 17A2; BioXcell) and anti‐CD28 (10 μg/mL; clone 37.51; BioXcell) in the presence of blocking anti‐mouse IL‐4 and anti‐mouse IFN‐g (10 μg/mL each; clones 11B11 and XMG1.2; BioXcell). 24 h later, cultures were exposed to viral supernatant for 1 h (centrifuged at 2200 rpm, 18°C), then cultured for a further 24–48 h in the presence of mouse IL‐27 (10 ng/mL; R&D Systems), anti‐mouse IL‐4 and anti‐mouse IFN‐g. Cells were then collected from culture plates and processed for cytometry or cell sorting. All cultures were in RPMI‐1640 medium supplemented with 10% fetal calf serum, 1% sodium pyruvate, 1% non‐essential amino acids, 10 mM HEPES, 0.1% β‐Mercaptoethanol, 100 U/mL penicillin and 100 mg/mL streptomycin.

### 2.5 Cell Counts and Flow Cytometry

For surface antigens, cells were stained and washed in phosphate‐buffered saline supplemented with 0.5% bovine serum albumin and 0.1% sodium azide. For phospho‐STAT3, cells were first pulsed with 10 ng/mL IL‐27 for 1 h, then fixed with 2% formaldehyde, permeabilized with 100% methanol and stained with fluorochrome labelled anti‐human/mouse pY703 STAT3 (Clone 4/P‐STAT3; BD Biosciences) together with relevant surface markers. Fluorochrome labelled antibodies were purchased from Thermo‐Fisher, BD Biosciences, or Biolegend. Dead cells were excluded using Live/Dead Aqua (Invitrogen) or Zombie NIR (Biolegend).

Mouse blood was collected from the retro‐orbital sinus into Eppendorf tubes in the presence of 5 mM ethylenediaminetetraacetic acid (EDTA, Sigma). Complete blood counts (CBC) were performed using an Element HT5 veterinary haematology analyser (Heska Corporation, Loveland, CO) for white blood cells (WBC), neutrophils (NEU), lymphocytes (LYM), monocytes (MON), red blood cells (RBC), haemoglobin (HGB) and platelets (PLT). Blood cells were incubated in Ammonium‐Chloride‐Potassium (ACK) lysing buffer twice of 10 min each to lyse RBCs and then stained with specific antibody mixtures for 30 min on ice for flow cytometry analyses. Monoclonal antibodies for murine CD4 (clone GK 1.5), CD8 (clone 53–6.72), CD11b (clone M1/70), CD44 (clone 1 M7), CD45R (RA3‐6B2), CD62L (clone MEL‐14), CD95 (Fas, clone SA637H8), F4/80 (clone BM48) and Ly‐6G (clone 1A8) were all from Biolegend (San Diego, CA), while Annexin V and the apoptosis detection kit were from BD Biosciences (Franklin Lakes, NJ). Antibodies were conjugated to fluorescein isothiocyanate (FITC), phycoerythrin (PE), PE‐cyanin 5 (PE‐Cy5), PE‐Cy5.5, PE‐cyanin 7 (PE‐Cy7), allophycocyanin (APC), APC‐Cy7, pacific blue (PB), brilliant violet 421 (BV421) or Alexa Fluor 647. Stained cells were acquired and analysed using a Cytek Aurora flow cytometer and associated software (Cytek Biosciences Inc., Fremont, CA).

### 2.6 Total RNA Sequencing (RNA‐Seq) and Data Analysis

Viable, GFP+ CD4+ CD8a + − or GFP+ CD4‐ CD8a + T cells were sorted 24–48 h post‐transduction (> 95% purity). 2–4 biological replicates were collected for each experimental group with similar numbers of cells per replicate (20–200 × 10^3^ per replicate group). These were lysed in Trizol reagent and total RNA purified by phenol‐chloroform extraction with GlycoBlue as co‐precipitant (7–15 μg per sample; Life Technologies). Poly(A) + mRNA was then enriched by oligo‐dT‐based magnetic separation and single‐end read libraries prepared with NEBNext Ultra RNA Library Prep Kit (New England Biolabs).

Total RNA was extracted from T cells isolated from spleen from wild‐type and mutant mice and purified with RNeasy Plus Mini Kit (Qiagen, 74,134). Ribosomal RNA was removed from 1 μg of total RNAs and cDNA was synthesised using SuperScript III (Invitrogen). Libraries for sequencing were prepared according to the manufacturer's instructions with TruSeq Stranded Total RNA Library Prep Kit with Ribo‐Zero Gold (Illumina, RS‐122‐2301) and paired‐end sequencing was done with a NovaSeq 6000 instrument and NextSeq 2000 (Illumina).

mRNA‐seq reads were aligned to mouse genome build mm10 with *tophat2*, assembled with *cufflinks*, and gene‐level counts compiled with *featureCounts*. To minimise normalisation artefacts, genes failing to reach an empirically defined count threshold were purged using *htsfilter*. 12–14 × 10^3^ genes were typically recovered post filtering, regardless of genotype or experimental group. Counts were normalised and differentially expressed genes (DEG) called by quasi‐likelihood F testing using *edgeR*. DEG call denotes > 2 fold pairwise changes and Benjamini‐Hochberg (BH) adjusted *p* value < 0.05. Transcripts per million (TPM) were compiled with *edgeR*. *clusterprofiler* was used for geneset enrichment analysis (GSEA) or hypergeometric testing (HGT) against KEGG, GO or Molecular Signatures (MSigDB) databases.

Total RNA‐seq read quality control was done using Trimmomatic [44] (version 0.36) and STAR RNA‐seq [45] (version STAR 2.5.4a) using paired‐end mode was used to align the reads (mm10). HTSeq [46, 47] was to retrieve the raw counts and subsequently, R (https://www.R‐project.org/), Bioconductor [48] and DESeq2 [49] were used. Additionally, the RUVSeq [50] package was applied to remove confounding factors. The data were pre‐filtered excluding *Rik*, *Gm* and *Mir* genes. Genes were categorised as significantly differentially expressed with log2 fold change > 0.5 or < −0.5 and adjusted *p*‐value (*p*_Adj_) < 0.05 corrected for multiple testing using the Benjamini‐Hochberg method were considered significant and then conducted gene enrichment analysis (Metascape, https://metascape.org/gp/index.html#/main/step1). The visualisation was done using dplyr (https://CRAN.R‐project.org/package=dplyr) and ggplot2 [51].

### 2.7 Chromatin Immunoprecipitation Sequencing (ChIP‐Seq) Data Analysis

Quality filtering and alignment of the raw reads was done using Trimmomatic [44] (version 0.36) and Bowtie [52] (version 1.3.1), with the parameter ‘‐m 1’ to keep only uniquely mapped reads, using the reference genome mm10. Picard tools (Broad Institute. Picard, http://broadinstitute.github.io/picard/ 2016) was used to remove duplicates; subsequently, Homer [53] (version 5.1) and deepTools [54] (version 3.5.4) software was applied to generate bedGraph files and normalise coverage separately. Integrative Genomics Viewer [55] (version 2.14.1) was used for visualisation. Macs [56] (version 2.2.7.1) peak‐finding algorithm was used to identify regions of ChIP‐seq enrichment over the background, utilising input files. For visualisation, the total reads number of mapped results in each sample was normalised to 10 million and background signals of < 5 were eliminated. To identify high‐confident STAT protein binding peaks, we defined the STAT3 and STAT5B binding peaks which contain at least one GAS motif (5′‐TTCNNNGAA‐3′) within the peak region using Macs, Homer and BEDTools [57] (version 2.31.1) software. The Integrative Genomics Viewer (IGV) tools were used for ChIP‐seq signal visualisation.

### 2.8 In Silico Structural Analysis of STAT3^D661^ Variants

The human STAT3β sequence (UniProt P40763‐3) [58] was used for structural modelling. Wild‐type proteins and variants (D661Y, D661V, D661H, D661N and K658N) were predicted with AlphaFold3 [59, 60] for both the full‐length protein (residues 1–722) and the SH2‐TAD region (residues 583–722). Ten models were generated per construct. Dimerization interfaces were classified as NTD‐, SH2‐TAD‐ or DBD‐mediated, and local hydrogen‐bond networks involving residues K658, D661, T663, N664 and I665 were examined. All structures were aligned and visualised in PyMOL (version 3.1; Schrödinger LLC, New York, NY, USA).

### 2.9 In Silico Pathogenicity Prediction

Multiple computational tools were employed to assess the potential pathogenicity of STAT3 variants. AlphaMissense [61] was used to predict the functional impact of mutations, with scores ranging from 0 (benign) to 1 (pathogenic). The Combined Annotation Dependent Depletion (CADD) [62] tool was utilised to estimate the deleteriousness of variants, where PHRED scores above 20 indicate potentially damaging effects. The Rare Exome Variant Ensemble Learner (REVEL) [63] scores, which range from 0 to 1, were calculated to assess the probability of pathogenicity. Additionally, PolyPhen‐2 [64] analysis was performed to predict the possible impact of amino acid substitutions on protein structure and function, with scores ranging from 0 (benign) to 1 (probably damaging).

### 2.10 Statistical Analyses

All samples that were used for CBC, FACS and RNA‐seq were randomly selected, and blinding was not applied. For comparison of samples, data were presented as standard deviation in each group and were evaluated with an unpaired two‐tailed *t*‐test with Welch's correction using PRISM GraphPad (version 10.1.1). Statistical significance was obtained by comparing the measures from the wild‐type or control group and each mutant group. A value of *p < 0.05, **p < 0.001, ***p < 0.0001, ****p < 0.00001 was considered statistically significant. ns, no significant.

### 2.11 Data Availability Statement

The RNA‐seq data generated in this study were uploaded in the Gene Expression Omnibus (GEO) under GSE3009953. RNA‐seq and ChIP‐seq of STAT5B^Y665F^ mutant mice were obtained under GSE276311 and GSE276308 [65]. ChIP‐seq data for STAT3 was downloaded from GSE217376 [66].

## 3 Results

Aspartic acid at position 661 (D661) is the most frequently mutated residue in the STAT3 SH2 domain, with 156 cases reported in the COSMIC database (Table 1) and the STAT3^D661Y^ and STAT3^D661V^ variants consistently associated with blood cancers in the gnomAD and COSMIC databases. In contrast, the STAT3^D661H^ and STAT3^D661N^ variants are either very rare or not present in these datasets. SH2 domain variants, including the D661 variants, are of particular interest because of the high conservation of this domain across vertebrate species (Figure S1). To evaluate the potential pathogenicity of these four STAT3^D661^ variants, we conducted comprehensive in silico analyses using structural prediction and multiple state‐of‐the‐art computational modelling tools (Table 1). Pathogenicity was evaluated using the NIH ClinVar database and additional predictive algorithms. ClinVar classifies D661Y and D661V as variants of uncertain significance, whereas D661H and D661N have not been previously reported in that database. AlphaMissense [61] predicted a severe functional impact for all four STAT3^D661^ variants, assigning scores between 0.970 and 0.995 and classifying them as pathogenic. PolyPhen‐2 [64] predicted all four variants to be probably damaging, with scores ranging from 0.967 to 0.999, reflecting a high degree of similarity in their predicted effects. Notably, REVEL (Rare Exome Variant Ensemble Learner) [63] analysis yielded scores of 0.708 for STAT3^D661Y^ and 0.718 for STAT3^D661V^, indicating a higher likelihood of pathogenicity for these two variants.

**TABLE 1:** Potential clinical pathogenicity of STAT3^D661^ mutations.

Gene	AA substitution	rsID	AllofUS	gnomAD	COSMIC	ClinVar	In silico pathogenicity score		
			Allele frequency	Allele frequency	Patient #	Clinical significance	AlphaMissense	PolyPhen2	REVEL
*STAT3*	D661Y	rs747639500	7.00E‐06	2.48E‐06	130	Uncertain significance	0.994	0.998	0.708
	D661V	rs2081519463	0	1.24E‐06	19	Uncertain significance	0.992	0.998	0.718
	D661H	—	0	—	5	—	0.995	0.999	—
	D661N	—	0	—	2	—	0.970	0.967	—

### 3.1 STAT3^D661^ Variants Reshape SH2 Domain Structure and Promote Dimerization

**FIGURE 1:**
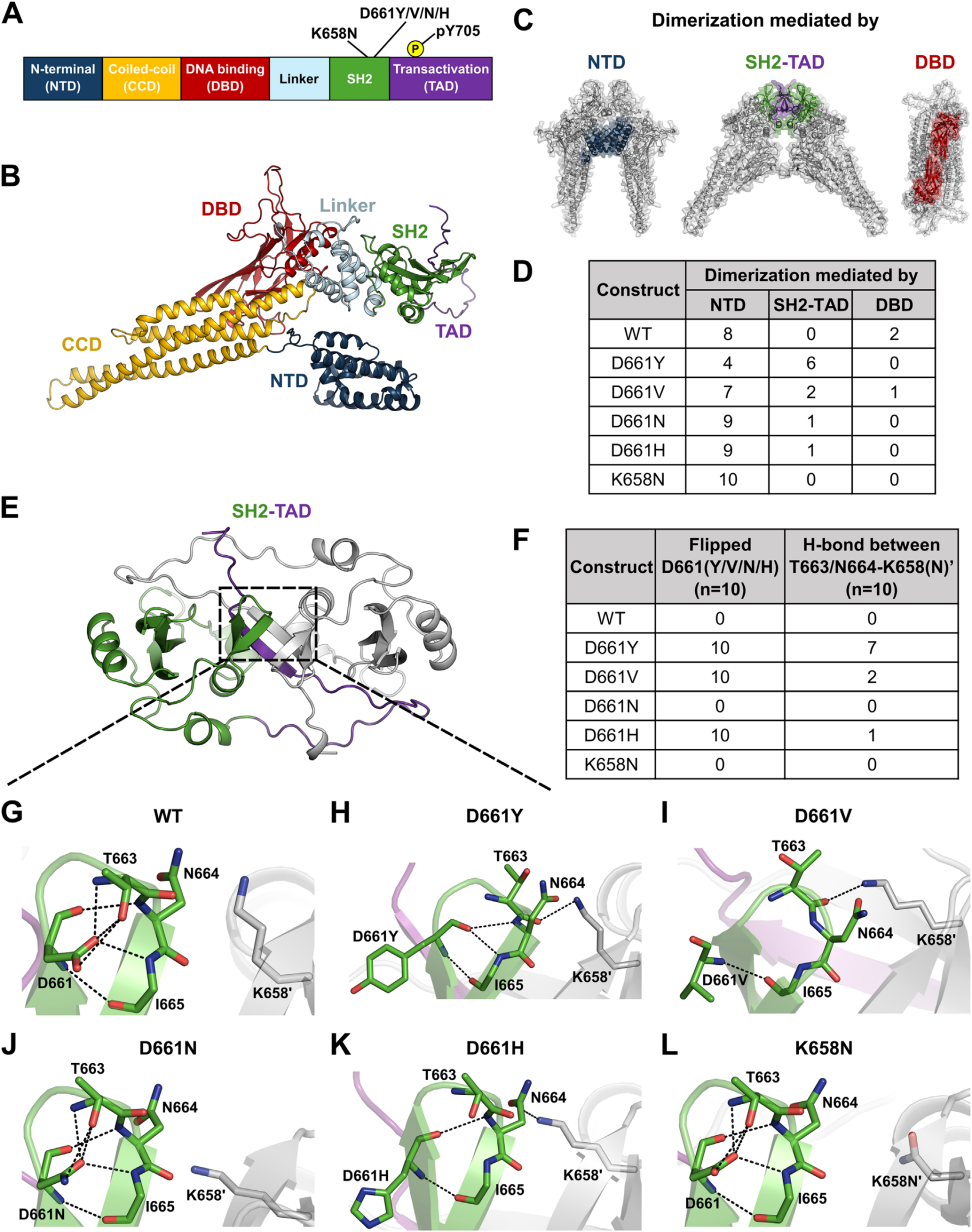
In silico structural analysis of STAT3^D661^ and STAT3^K658N^ mutations. (A) Domain architecture of STAT3. D661 mutations, K658N and the phosphorylation site Y705 are highlighted. (B) Overall structure of an AF3‐predicted STAT3 wild‐type monomer, with domains coloured as in (A). (C) Schematic representation of the three distinct predicted STAT3 dimer conformations. The domains mediating dimerization are coloured as in (A). (D) Summary table of the ten predicted dimer models, categorised by the domains mediating dimerization. (E) Overall structure of the SH2‐TAD wild‐type dimer. One subunit is coloured in green and purple, and the neighbouring molecule is in grey. The loop containing residues D661 and K658 is highlighted. (F) Table summarising the conformations of D661 and the presence of hydrogen bonds between N664 and K658 (N)'. Apostrophes indicate residues from the neighbouring molecule. (G‐L) Local conformations of the loop containing D661 and K658' for (G) WT, (H) D661Y, (I) D661V, (J) D661N, (K) D661H and (L) K658N. Hydrogen bonds are depicted as black dashed lines.

To assess the structural impact of D661 variants, located within the Src homology 2 (SH2) domain of STAT3 [67] (Figure 1A,B), we employed AlphaFold3‐based in silico modelling [59, 60], which allows prediction of multiple conformational states [68]. First, we modelled unphosphorylated dimers with both the wild‐type and variant proteins. For these predictions, we used the STAT3β isoform sequence, whose domain structures have been experimentally validated [67, 69, 70]. The modelled variants included D661Y/V/H/N, as well as the nearby somatic GOF variant K658N [27] for comparison.

Phosphorylation at Y705 promotes STAT3 dimerization [71]. Thus, we also modelled effects of D661 variants on dimerization. Three distinct conformations were observed for full‐length dimer modelling, with dimerization mediated by the N‐terminal domain (NTD), the SH2‐transactivation domains (SH2‐TAD) or the DNA‐binding domain (DBD) (Figure 1C). These conformations closely resemble previously determined STAT3 structures in the parallel, free (PDB: 3CWG) [72], parallel, double‐stranded DNA (dsDNA)‐bound (PDB: 1BG1) [67] and antiparallel (PDB: 6TLC) [73] states, respectively. Ten models were predicted per construct, and the domain involved in dimerization was analysed (Figure 1D). For the wild type, eight models dimerized via the NTD and two via the DBD. In contrast, the D661Y shifted the distribution of predicted models toward SH2‐TAD‐mediated dimerization (six models). Similar shifts were observed for D661V (two models), D661N (one) and D661H (one). By contrast, all ten models of K658N dimerized exclusively via the NTD. These results suggest that D661 mutations promote SH2‐TAD‐mediated dimerization and induce the conformational change toward the dsDNA‐bound state.

To further dissect local effects, we further modelled only the SH2‐TAD (residues 583–722) in the unphosphorylated state (Figure 1E). Ten structures were predicted for each construct and examined for key residue interactions (Figure 1F). The overall structures of the mutants were largely unchanged, with root‐mean‐square‐deviations (RMSD) of less than 0.8 Å relative to WT. However, local changes of the hydrogen‐bonding network were observed. D661 and K658 are located on a loop spanning residues G666‐K679 at the SH2 dimer interface. In the WT, D661 forms extensive hydrogen bonds with T663, N664 and I665, thereby stabilising the loop structure (Figure 1G). This positioning also prevents N664 from forming a hydrogen bond with the neighbouring K658' (apostrophe denotes the neighbouring molecule). All predicted WT models preserved this conformation.

In D661Y, however, this stabilising interaction was lost (Figure 1H). In nine out of ten models, D661Y flipped away from T663, allowing the main‐chain carbonyl of T663 to shift toward K658' and form a new hydrogen bond, potentially strengthening SH2‐SH2 domain interactions. Similar conformational changes were observed for D661V, with the T663 carbonyl group repositioned closer to K658' (Figure 1I). By contrast, D661N retained hydrogen bonds similar to WT (Figure 1J), indicating minimal structural impact. D661H also flipped away from T663, but T663 retained a WT‐like orientation, while N664 shifted by ~2 Å to form a hydrogen bond with K658', suggesting partial stabilisation of the SH2 dimer (Figure 1K). In K658N, D661 maintained its WT orientation (Figure 1L). However, substitution with the shorter side chain for K658N' prevented hydrogen bond formation with N664, likely weakening the dimerization interaction.

Taken together with the relative abundance of molecular states and hydrogen bond networks indicates a varying impact of these mutations on STAT3 structure. D661Y is predicted to have the strongest effect, followed by D661V, while D661N and D661H are milder. Unlike the D661 substitutions, K658N did not promote SH2‐TAD dimerization and instead favoured NTD‐mediated dimerization, highlighting its distinct structural impact.

To explain the graded phenotypes of the D661 variants, we compared each substitution to the biochemical properties of wild‐type aspartate. Aspartate is negatively charged and polar; replacement with tyrosine or valine represents a major shift—tyrosine introduces a bulky aromatic ring, and valine is hydrophobic and neutral [65, 74]. In contrast, histidine and asparagine retain polarity and hydrogen‐bonding potential, making them more similar to aspartate. This relative divergence (D661Y > D661V > D661H > D661N) parallels our structural modelling and correlates with phospho‐STAT3 accumulation, transcriptional activity and immune remodelling, indicating that biochemical distance from Asp directly predicts GOF strength.

Prior SH2‐domain studies further support the functional importance of D661. Liu et al. [75] defined D661 as part of the conserved bc5 position shaping the pY + 3 specificity pocket, while Chen et al. [76] showed that substitutions at this site decrease electronegativity and alter aromatic packing, with perturbation magnitude correlating with STAT3 hyperactivity. Together with our modelling, transcriptomics and in vivo data, these findings establish a mutational gradient in which D661Y and D661V produce the strongest disruption, followed by D661H and D661N, highlighting D661 as a structurally privileged regulator of STAT3 activation.

### 3.2 STAT3^D661^ Variants Exhibit a Hierarchy of Transcriptional Effects in T Cells In Vitro

**FIGURE 2:**
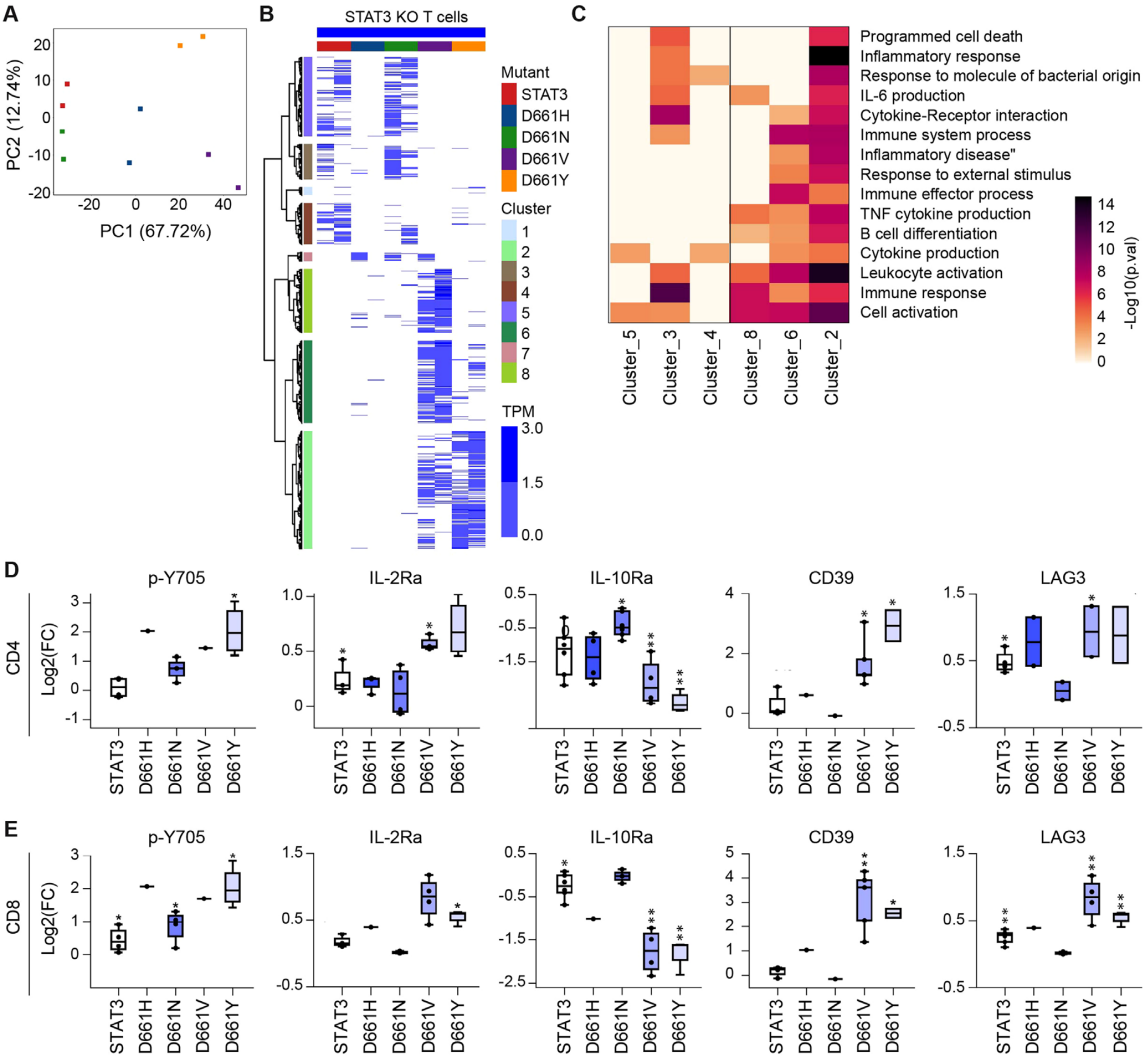
Transcriptomic programs activated by wild‐type and STAT3D661 mutants in Stat3‐null T cells. (A) Principal component analysis (PCA) of transcriptomic profiles from Stat3‐null T cells transduced with retroviral vectors encoding STAT3^D661H^, STAT3^D661N^, STAT3^D661V^ or STAT3^D661Y^ compared to wild‐type STAT3 (RNA‐seq, *n* = 2). (B) Heatmap of hierarchical clustering analysis of DEGs. (C) Heatmap depicting cluster‐specific gene expression patterns and their significantly enriched GO categories. (D–E) Bar plots showing surface expression of canonical STAT3 target markers in CD4^+^ and CD8^+^ T cell populations reconstituted with wild‐type STAT3 or STAT3^D661^ variants (*n* = 4).

To assess downstream consequences for STAT3^D661^ mutations, we assessed transcriptomes in T cells expressing either wild‐type (WT) STAT3 or the variants (STAT3^D661H^, STAT3^D661N^, STAT3^D661V^ or STAT3^D661Y^) (Figure 2, Table S1). Briefly, naïve T cells were purified from lymph nodes and spleens of mice carrying two *Stat3* floxed alleles and a *cd4‐Cre* transgene resulting in the deletion of *Stat3* in CD4 and CD8 T cells. *Stat3*‐null T cells were transduced with STAT3‐expressing retroviral vectors (WT or variant), then cultured in the presence of IL‐27, a potent STAT3 activating cytokine and processed for ‘bulk’ RNA‐seq. RNA‐seq confirmed an approximately 98% reduction of Stat3 mRNA in *Stat3* ^−/−^ T cells relative to WT controls, validating efficient deletion of the *Stat3* locus and establishing a null background for functional reconstitution. Retroviral expression of either WT or mutant STAT3 restored *Stat3* mRNA expression to near‐WT levels, ensuring comparable baseline transcriptional input across conditions.

Transcriptome analysis revealed that STAT3^D661N^ induced only 14 differentially expressed genes (DEGs) compared to WT STAT3, consistent with a functionally silent mutation. By contrast, STAT3^D661V^, STAT3^D661Y^ and STAT3^D661H^ induced 1357, 761 and 680 DEGs, respectively (Table S1), confirming their gain‐of‐function (GOF) activity (Figure 2A,B). Clustering analysis further demonstrated that DEGs enriched in STAT3^D661V^, STAT3^D661Y^ and STAT3^D661H^ (clusters 2, 6 and 8) were strongly associated with cytokine signalling and immune activation pathways, whereas DEGs enriched in WT and STAT3^D661N^ (clusters 3, 4 and 5) showed markedly weaker association with immune‐related GO term enrichment compared to the three mutations (Figure 2C). Notably, pro‐inflammatory pathways were particularly enriched in clusters 2 and 6, suggesting that these mutations may drive transcriptional programs linked **to** autoimmune components frequently observed in T cell malignancies such as T‐LGLL [77]. Taken together, these results demonstrate that STAT3^D661V^, STAT3^D661Y^ and STAT3^D661H^ function as activating GOF mutations, with STAT3^D661V^ and STAT3^D661Y^ showing stronger transcriptional effects than STAT3^D661H^. By contrast, STAT3^D661N^ behaves as a functionally silent mutation, closely resembling WT STAT3. Functionally, retroviral expression of STAT3^D661H^, STAT3^D661V^ and STAT3^D661Y^, but not STAT3^D661N^, induced surface expression of canonical STAT3 target genes [78, 79, 80], including IL‐2Rα (CD25), IL‐10Rα (CD210), CD39 (ENTPD1) and LAG‐3 (CD223) in both CD4^+^ and CD8^+^ T cells (Figure 2D,E).

### 3.3 Severe GOF Variants Impair Viability, Whereas STAT3^D661H^ Alters Development and Immune Homeostasis

Complete genetic deletion of STAT3 in mice results in early embryonic lethality (E3.5–E4.5) due to defects in mesoendoderm and mesoderm migration [81, 82, 83]. In contrast, STAT3β knock‐in mice, which differ from STAT3α by seven unique C‐terminal residues arising from alternative splicing, are viable and largely redundant with STAT3α [84]. This distinction provides context for understanding the lethality observed in our STAT3^D661^ GOF knock‐in models. Additionally, C‐terminal truncation of STAT3 can occur in myeloid cells via protease‐mediated cleavage, which may influence functional readouts in immune contexts.

To investigate the in vivo consequences of STAT3^D661^ variants, we introduced three variants into the mouse genome using CRISPR/Cas9. Ultimately, we succeeded in generating mouse lines carrying the STAT3^D661H^ variant, but not the STAT3^D661Y^ and STAT3^D661V^ variants. Introduction of the STAT3^D661H^ mutation yielded 15 pups, four of which carried the correct hemizygous variant and two the correct homozygous variant (Table 2; Figure S2A). Introduction of the STAT3^D661V^ mutation yielded 14 pups, and the two carrying the correct hemizygous variant died within 2 weeks after birth. The sole pup derived from the STAT3^D661Y^ targeting died within 1 week after birth and its mutational status is unknown. The postnatal lethality suggests that the STAT3^D661Y^ and STAT3^D661V^ mutations may significantly compromise viability. These observations agree with the in vitro modelling data suggesting a more prominent impact of the STAT3^D661Y^ and STAT3^D661V^ variants.

**TABLE 2:** The number of mutant founder mice carrying *Stat3* point mutations.

Mutation	Number of embryos injected with sgRNA	Number of 2‐cell embryos implanted into surrogate mothers	Number of mice born	Number of founder mice carrying mutations	Viability
D661Y	388	296	1	0	Early death (1–2 weeks)
D661V	1143	829	14	2 (het)	
D661H	964	375	15	13 (4 het/2 homo)	Survival

The STAT3^D661H^ founder mice exhibited delayed body growth (Figure S2B). Although impaired growth is observed in multiple STAT3 GOF contexts [27, 31, 33, 35, 36, 85], we did not measure hepatic *Igf1* expression or serum IGF‐1 here, so a direct causal link to GH–IGF‐1 axis dysfunction cannot be inferred from the present data. Breeding of STAT3^D661H^ hemizygous mice did not yield the expected Mendelian ratio of 25% homozygous and 50% hemizygous offspring. Among 21 pups from six matings of hemizygous males and females, no homozygous pups were recovered, and only one‐fourth of the offspring were hemizygous. Furthermore, STAT3^D661H^ hemizygous mothers were unable to adequately nurse their offspring, resulting in pups that were hairless, exhibited severely stunted growth and approximately 50% died prematurely within a few months.

### 3.4 STAT3^D661H^ Alters T Cell Homeostasis and Subtype Populations

**FIGURE 3:**
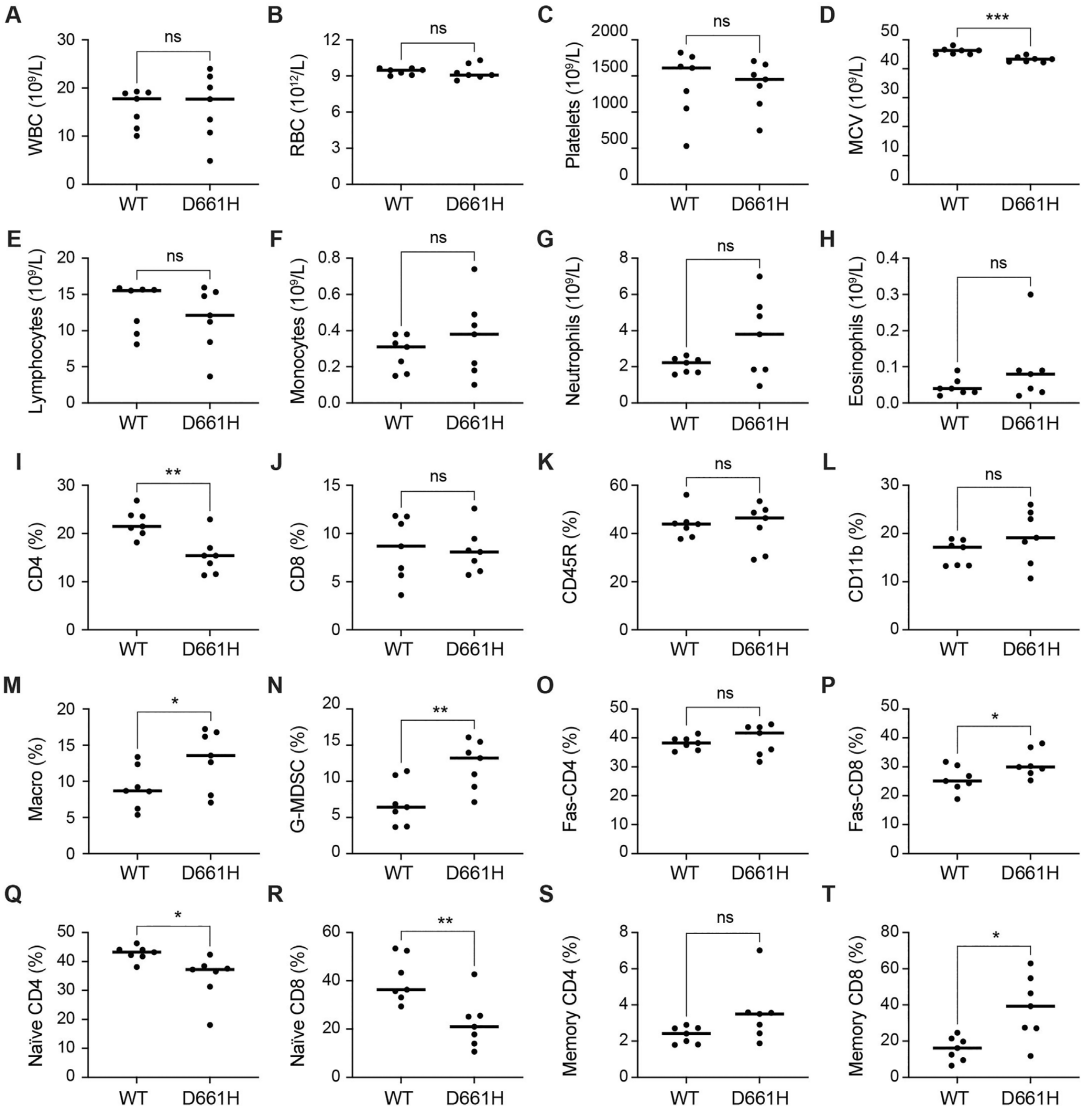
Haematological parameters and altered immune phenotypes of STAT3^D661H^ mutant mice. (A–H) Peripheral blood cell counts in 3‐month‐old adult wild‐type and STAT3^D661H^ mutant mice. Results are shown as the median from independent biological replicates (*n* = 7). *p*‐values were calculated using an unpaired two‐tailed t‐test with Welch's correction. *p < 0.05, **p < 0.01, ***p < 0.001. (I–T) Quantification of immune cell subpopulations identified by flow cytometry.

To assess the impact of the STAT3^D661H^ variant on the immune system, we analysed peripheral blood from three‐month‐old STAT3^D661H^ hemizygous mice. Hemizygous STAT3^D661H^ animals exhibited reduced body size and smaller spleens compared to wild‐type controls (Figure S2B,C), yet total blood cell counts were largely comparable, except for a significant decrease in mean corpuscular volume (MCV) (Figure 3A–H). The reduction in MCV indicates smaller average red blood cell size, a feature typically associated with microcytic anaemia (Figure 3D).

Analysis of lymphocyte subsets revealed a marked reduction in total CD4^+^ T cells in STAT3^D661H^ hemizygous mice, whereas CD8^+^ T cells, B cells (CD45R^+^) and myeloid‐lineage cells, including neutrophils (CD11b^+^), were maintained at levels comparable to wild‐type controls (Figure 3I–L). Within the CD4^+^ T cell compartment, the reduction was primarily restricted to naïve CD4^+^ T cells, while memory CD4^+^ T cells remained unchanged. In contrast, although total CD8^+^ T cell numbers were preserved, STAT3^D661H^ mice exhibited a significant decrease in naïve CD8^+^ T cells accompanied by an expansion of memory CD8^+^ T cells (Figure 3Q–T). Interestingly, STAT3^D661H^ mice showed a selective increase in CD11b^+^Ly6G^+^ granulocytic myeloid‐derived suppressor cells (G‐MDSCs), alongside macrophages, suggesting a potential compensatory expansion of immunosuppressive myeloid subsets (Figure 3M,N). Finally, apoptosis analysis by Fas staining revealed no differences in CD4^+^ T cells but a significant reduction in apoptosis among CD8^+^ T cells from STAT3^D661H^ mice (Figure 3O,P), indicating that this mutation may confer a survival advantage specifically within the CD8^+^ T cell compartment.

### 3.5 STAT3^D661H^ Variant Enhances Cytokine Signalling and Immune Activation Programs

**FIGURE 4:**
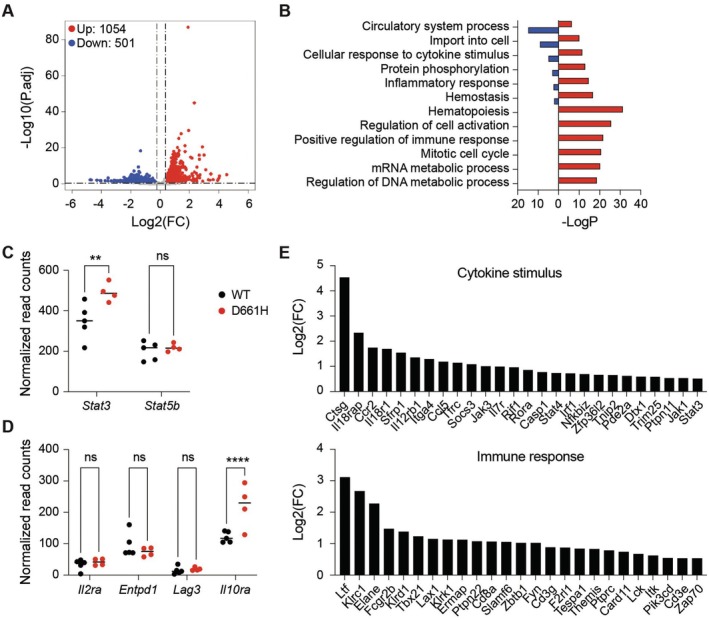
Transcriptomic profiling of splenic T cells from STAT3^D661H^ mutant mice reveals altered immune gene programs. (A) Volcano plot depicting differentially expressed genes (DEGs) between STAT3^D661H^ and wild‐type (WT) splenic T cells. Genes with an adjusted *p*‐value (*p*_adj_) < 0.05 and a log2 fold change (FC) > 0.5 or < −0.5 are shown in red (upregulated) and blue (downregulated), respectively; non‐significant genes are shown in grey. Total numbers of upregulated and downregulated genes are indicated. (B) Gene ontology analysis of DEGs reveals significant enrichment in categories related to T cell activation and immune system processes in STAT3^D661H^ mutants compared to WT. (D) Dot plot showing normalised RNA‐seq read counts for *Stat3*, *Il2ra*, *Entpd1*, *Lar3* and *Il10ra* mRNA, highlighting its autoregulatory induction in STAT3^D661H^ mutants. Data represent mean ± SEM of biological replicates (*n* = 4). Statistical significance was assessed using the Benjamini–Hochberg adjusted *p*‐value. ***p < 0.001, ****p < 0.0001. Stat5b expression is shown as a control. (E) Bar graphs displaying relative log2 fold changes in representative genes involved in cytokine stimulus response and positive regulation of immune responses.

To further explore downstream consequences for the STAT3^D661H^ variant, we performed bulk RNA‐sequencing on ‘resting’ splenic T cells isolated from STAT3^D661H^ and wild‐type mice. Transcriptomic profiling revealed 1054 significantly upregulated differentially expressed genes (DEGs) compared to wild‐type controls (Figure 4; Table S2), including 210 genes known to harbour STAT3 binding sites [4]. These DEGs were significantly enriched for gene ontology categories related to immune system processes and activation (Figure 4A,B; Table S2). A notable finding was the upregulation of *Stat3* itself (Figure 4C), consistent with its known autoregulatory role in driving transcriptional programs, such as those observed in T cells [86, 87]. The *Stat5b* gene is also under autoregulatory control [88] but its expression was not induced by STAT3^D661H^ (Figure 4C), suggesting gene‐specific activation of the STAT3^D661H^ variant. Moreover, surface protein genes known as canonical STAT3 targets—including *Il2rα*, *Entpd1*, *Lag3* and *Il10ra*—showed increased median expression levels in T cells from STAT3^D661H^ mice, with IL‐10Rα exhibiting a statistically significant upregulation (Figure 4D). To further assess the functional impact of the STAT3^D661H^ variant on immune regulation, we focused on two key gene sets: response to cytokine stimulus and immune response. Genes involved in cytokine signalling pathways—including members of the JAK–STAT cascade—and immune activation genes were markedly upregulated in STAT3^D661H^ T cells (Figure 4E; Table S2), indicating enhanced immune transcriptional programming.

### 3.6 STAT3^D661H^ and STAT5B^Y665F^ Co‐Regulate Core Immune Genes but Maintain Distinct Transcriptional Signatures

**FIGURE 5:**
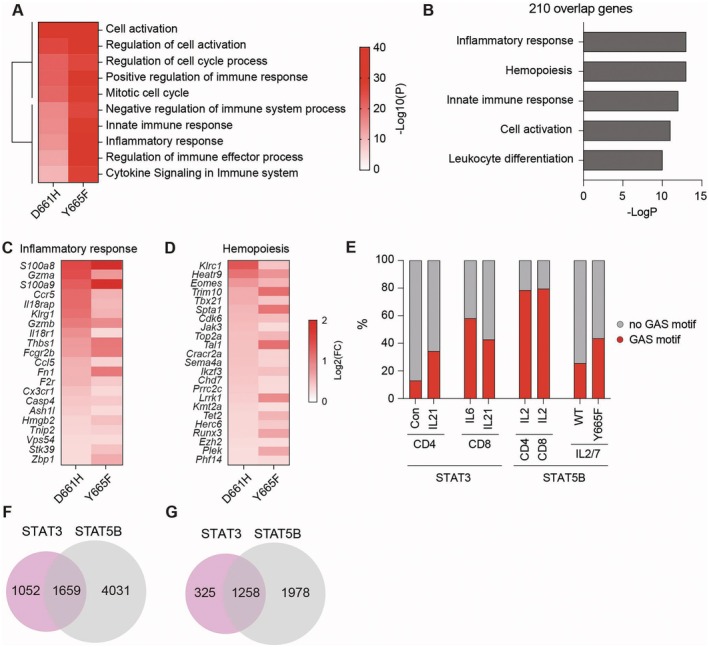
Comparative analysis of transcriptional programs in T cells from STAT3^D661H^ and STAT5B^Y665F^ mutant mice. (A) Heatmap showing genes significantly upregulated in STAT3^D661H^ and STAT5B^Y665F^ mutants, with corresponding Gene Ontology (GO) terms enriched in each group. (B) Functional enrichment analysis of 210 genes commonly upregulated in both mutants, highlighting pathways related to immune activation. (C, D) Heatmaps illustrating log2 fold changes of upregulated genes associated with inflammatory responses (C) and haematopoiesis (D) in each mutant group. (E) Proportions of STAT3 and STAT5B chromatin‐binding peaks containing the canonical GAS motif, as identified by ChIP‐seq following cytokine stimulation. (F–G) Venn diagrams showing the number of GAS motif–containing STAT3 and STAT5B binding peaks (F) and the overlap of predicted target genes proximal to these peaks (G).

We previously reported that STAT5B^Y665F^, a gain‐of‐function (GOF) variant, leads to enhanced STAT5 activity and immune gene expression in T cells [65], specifically the induction of genes significantly enriched in immune activation pathways compared to wild‐type controls. Building on these findings, we sought to compare the transcriptional programs of STAT3^D661H^ and STAT5B^Y665F^ to identify shared and distinct immune‐regulatory gene networks mediated by these two STAT family members. Transcriptome profiling revealed that both variants significantly upregulated immune‐related genes compared to wild‐type controls, with STAT5B^Y665F^ and STAT3^D661H^ inducing 1776 and 1054 genes, respectively. Gene ontology analysis highlighted shared enrichment in pathways such as cell activation, cytokine signalling and immune effector responses (Figure 5A; Table S2). Notably, STAT5B^Y665F^ exhibited a broader and more potent effect on immune gene expression than STAT3^D661H^. Among the DEGs, 210 were commonly regulated by both variants (Table S2), with significant enrichment in inflammatory response, haematopoiesis, innate immunity and leukocyte activation (Figure 5B). STAT3^D661H^ and STAT5B^Y665F^ both increased expression of key inflammatory genes such as *Gzma*, *Gzmb*, *S100a8* and *Il18r1* (Figure 5C), as well as haematopoietic regulators including *Klrc1*, *Jak3* and *Runx3* (Figure 5D).

To determine whether the observed transcriptional changes were directly mediated by the STAT3 and STAT5B variants, we analysed ChIP‐seq datasets from T cells stimulated with IL‐2, IL‐6 or IL‐21 [65, 66]. Genome‐wide binding analysis revealed that STAT5B exhibited broader occupancy than STAT3, particularly at canonical GAS motifs (TTCnnnGAA) (Figure 5E; Table S3). Among 2711 STAT3 peaks and 5690 STAT5B peaks containing GAS motifs, 1659 were shared. This translated to 1258 shared target genes among 1583 STAT3‐associated and 3236 STAT5B‐associated genes (Figure 5F,G). Notably, both STAT3 and STAT5 binding was observed at promoter and enhancer regions of key STAT target genes, such as *Cish, Lfng* and *Socs2* (Figure S3). These findings suggest that while STAT3 and STAT5B co‐regulate a large set of immune‐related genes, they also have distinct elements within their transcriptional programs. Collectively, these results suggest that STAT3 and STAT5B not only share common immune gene targets but also exhibit distinct transcriptional regulatory mechanisms, reflecting their unique roles in shaping immune responses.

## 4 Discussion

Aspartic acid 661 (D661) is the second most common frequent STAT3 mutational hotspot, with four variants (D661Y, D661V, D661H and D661N) reported in hematologic malignancies. Using AlphaFold3‐based structural modelling, pathogenicity predictors, reconstitution experiments in Stat3‐null T cells, and CRISPR/Cas9 mouse genetics, we demonstrate that substitutions at this conserved SH2‐domain residue produce graded biochemical and physiological effects, following a consistent severity hierarchy (D661Y ≈ D661V > D661H > D661N).

In silico approaches (AlphaFold3, AlphaMissense, ClinVar, REVEL and PolyPhen‐2) revealed a complex picture of the variants' potential effects and were unable to fully distinguish variant strength, highlighting the limitations of computational prediction alone. Structural modelling indicated that D661 variants promote STAT3β dimerization through the SH2‐TAD interface and stabilise an active conformation, providing a mechanistic basis for the observed gradient of transcriptional activity.

AlphaFold3 modelling predicts enhanced pY–SH2 dimer interfaces in all D661 GOF variants, consistent with increased STAT3 activation. Because SH2 domains occur in approximately 116 human proteins—including STATs, SOCS, JAKs, adaptors (GAB1–3, GRB2) and E3 ligases (CBL/CBLB)—such strengthening may influence broader signalling networks, including TP53‐linked degradation pathways and RAS‐driven oncogenic cascades [76, 89, 90, 91, 92]. Although mitochondrial STAT3 was not examined here, increased SH2‐mediated stability could conceivably impact mitochondrial STAT3 function, representing a valuable direction for future work.

Growth impairment has been reported in multiple STAT3 GOF models, but its mechanistic basis remains unclear. While GH–GHR signalling activates STAT5B to induce Igf1 expression, reduced STAT5 levels allow STAT3 recruitment to the GHR–JAK2 complex and increases GH‐induced pY‐STAT3 [93, 94, 95]. Thus, strong GOF alleles could disrupt growth either indirectly through STAT5 imbalance or directly via altered SH2‐mediated engagement of GHR/JAK2. Dedicated endocrine assays will be required to test whether the D661 variants perturb GH–IGF signalling, and we now frame this as a future direction rather than a conclusion.

Notably, these computational predictions were consistent with in vitro and in vivo experiments: D661V and D661Y mutants exhibited stronger transcriptional activity in T cells (1357 and 761 DEGs, respectively) and produced more deleterious effects on mouse viability (early postnatal lethality for D661V/Y targeting) than the milder D661H (680 DEGs) and functionally silent D661N variants. The structural analyses revealed a gradient of variant severity (Y > V > H > *N*), and both the in vitro transcriptomic studies and the mouse genomic analyses supported this trend. The D661Y and D661V variants appear to stabilise SH2–SH2 interactions and favour a conformation associated with transcriptional competence, thereby facilitating cytokine‐independent dimerization and persistent target gene activation. This mechanism is consistent with the induction of canonical STAT3 targets and broad immune activation signatures in our reconstituted T cell RNA‐seq data.

The D661H variant produced intermediate structural perturbations and a milder transcriptional phenotype in vitro, which translated in vivo into survival of heterozygotes but with defects in growth, CD4^+^ T cell numbers, skewing of CD8^+^ subsets toward a memory phenotype, expansion of granulocytic MDSCs, and altered apoptosis of CD8+ cells. By contrast, D661N was essentially silent across assays, consistent with modest predicted structural perturbation. In the retroviral system, STAT3^D661H^ induced 680 DEGs compared to WT, classifying it as an activating GOF mutation. The mouse model showed 1054 upregulated DEGs in splenic T cells compared to WT. The in vivo induction of Stat3 itself in the STAT3^D661H^ T cells is consistent with the enhanced transcriptional activity predicted from the in vitro data and structural modelling. STAT3^D661H^ T cells show upregulation of Stat3 itself, consistent with autoregulatory loops that can amplify mutant activity; DEGs are enriched for cytokine signalling and immune activation pathways, and many upregulated genes overlap with loci bound by STAT3 in ChIP datasets.

Published models carrying GOF alleles (L387R, K392R, G421R, K658N, T716M) (Table S4) display a spectrum of immune dysregulation, including lymphadenopathy, splenomegaly, dermatitis, autoimmune cytopenias and in some cases perinatal lethality or reduced viability when homozygous. The STAT3^D661H^ hemizygous mice, like STAT3^K658N^ and STAT3^L387R^ hemizygous mice, also exhibited impaired viability and delayed body growth [85], suggesting that D661H confers a significant GOF phenotype. This severe outcome for homozygosity across multiple GOF mutations underscores the essential, dosage‐sensitive role of STAT3 in development. The nursing failure observed in STAT3^D661H^ hemizygous mothers is an additional complexity that aligns with the established role of STAT3 in regulating growth hormone and potentially other non‐haematopoietic functions, a feature often associated with GOF mutations. However, unlike several other GOF mutants that produce robust lymphoproliferation and obvious splenomegaly/dermatitis in heterozygotes (e.g., STAT3^G421R^, STAT3^K658N^) [27, 96], the STAT3^D661H^ mice did not show splenomegaly or overt skin disease at the ages examined, suggesting either a milder systemic phenotype or a requirement for environmental triggers/age to reveal full pathology. The early lethality of D661Y/V parallels the severity reported for certain other SH2 mutations (K658N) when expressed constitutively, reinforcing the concept that the biochemical properties of SH2‐altering mutations (i.e., propensity to drive constitutive dimerization) determine organismal tolerance.

Across STAT3 GOF alleles, organismal tolerance tracks closely with biochemical activation strength. DNA‐binding domain mutants (L387R, K392R, G421R) are viable at birth but later develop growth delay, immune infiltration and organomegaly [85, 96, 97]. SH2 variants span a wider severity range: mice homozygous for K658N are viable with partial penetrance of immune disease, while G656–M660del causes minimal pathology despite measurable GOF activity [27]. In contrast, D661Y and D661V were incompatible with germline viability, exceeding even several DBD mutants in developmental intolerance [32, 74, 98]. D661H produced a moderate phenotype—immune skewing with reduced naïve CD4^+^ and expanded memory CD8^+^ T cells—consistent with clinical observations. D661N was functionally silent. Together, these results support a continuum rather than a binary classification of STAT3 activation, in which stronger SH2‐activating alleles correlate with reduced viability.

While homozygous germline deletion of STAT3 in mice results in early embryonic lethality [82], STAT3 variants have generally a less profound impact on mouse development. Among the STAT3 variants investigated, D661H mice were viable in the heterozygous but not the homozygous state suggesting embryonic or perinatal death. Since wild type STAT3 is critical for embryonic development, an activated STAT3 could certainly have an adverse impact in the homozygous state. The few founder mice carrying the D661Y and D661V variants died perinatally and it was not possible to conduct molecular pathology. Drawing conclusions from founder mice is confounded by the possibility that one or both targeted alleles could be subject to complex deletions and the correct targeting would be mosaic in nature. Homozygosity of other variants, including L378R and G421R, also results in postnatal death. While postnatal death of Stat5a/b‐null mice likely results from haematopoietic defects [99] this does not appear to be the case with the STAT3 variants studied here.

Our comparative analysis with STAT5B^Y665F^ GOF data revealed overlapping yet distinct transcriptional programs: both mutants activate immune activation and cytotoxicity‐associated genes, but STAT5B^Y665F^ exhibits broader genomic occupancy and stronger transcriptional output. These differences likely arise from distinct cytokine receptor inputs (STAT3: IL‐6/IL‐10/IL‐21/gp130 family; STAT5B: IL‐2/IL‐7/IL‐15 and growth hormone pathways), differential cofactor recruitment and intrinsic DNA‐binding preferences/modulation. Clinically, the variant‐specific effects we observe are relevant because D661Y/V are enriched in patient cohorts, consistent with a model in which the strongest GOF alleles are selected during clonal expansion in hematologic disease but are deleterious when present constitutively in the germline.

## 5 Conclusion

Our integrated structural, cellular and genetic analyses establish a variant‐specific hierarchy of functional consequences for STAT3^D661^ substitutions and demonstrate that even closely related SH2 mutations can produce distinct transcriptional and physiological outcomes. These data refine our understanding of STAT3‐driven immune dysregulation and provide a framework for mechanistic and therapeutic studies of STAT3 GOF disorders.

## 6 Limitations

Several caveats temper our conclusions. First, AF3 models are static and represent a starting point for mechanistic hypotheses rather than definitive proof of altered dynamics or phosphorylation‐dependent behaviour. Second, we have used STAT3 (1–722) and not the full‐length STAT3 (1–770) for the AF3 analysis. Third, four RNA‐seq of splenic T cells was performed on mixed T cell populations with altered subset proportions; although we accounted for this limitation, single‐cell approaches would more precisely dissect cell‐intrinsic transcriptional reprogramming. Third, the absence of viable mice carrying the D661Y/V variants precluded direct in vivo comparison of these alleles; conditional or cell type–restricted expression systems may be necessary to study their tissue‐specific effects. Finally, sample sizes for some assays and ages were limited; longer‐term aging and immune challenge experiments could reveal progressive or context‐dependent phenotypes.

## Author Contributions

**Hye Kyung Lee:** conceptualization, investigation, writing – original draft, visualization, formal analysis, project administration, data curation, methodology, writing – review and editing. **Gyuhyeok Cho:** conceptualization, investigation, writing – original draft, writing – review and editing, visualization, methodology, formal analysis, software, data curation. **Jichun Chen:** investigation, writing – original draft, validation, formal analysis. **Aaron B. Schultz:** investigation, formal analysis. **Sung‐Gwon Lee:** conceptualization, investigation, formal analysis. **Chengyu Liu:** investigation, methodology, validation, writing – original draft, formal analysis. **Priscilla A. Furth:** conceptualization, investigation, writing – original draft, validation, writing – review and editing, formal analysis, project administration, supervision. **Neal S. Young:** funding acquisition, investigation, supervision. **Jungwook Kim:** funding acquisition, writing – original draft, project administration. **Alejandro Villarino:** conceptualization, investigation, funding acquisition, writing – original draft, formal analysis, project administration, supervision. **Lothar Hennighausen:** conceptualization, investigation, funding acquisition, writing – original draft, writing – review and editing, formal analysis, project administration, supervision, resources.

## Ethics Statement

All animals were housed and handled according to the Guide for the Care and Use of Laboratory Animals (8th edition) and all animal experiments were approved by the Animal Care and Use Committee (ACUC) of National Institute of Diabetes and Digestive and Kidney Diseases (NIDDK, MD) and the University Animal Care and Use Committee (University of Miami) and performed under the NIDDK animal protocol K089‐LGP‐23.

## Conflicts of Interest

The authors declare no conflicts of interest.

## Supporting information


**Figure S1:** Cross‐species conservation of STAT3 amino acid sequences, with a focus on the region surrounding D661.
**Figure S2:** STAT3 D661 mutations in the mouse genome. (A) Sanger sequencing chromatograms showing wild‐type (WT) and the introduction of SNPs resulting in the two missense mutations, STAT3D661V (D661V) and STAT3D661H (D661H) mutants. The red shade indicates the altered codons converting D661 to D661V and D661H, respectively. (B) Images of founder mice carrying STAT3D661H mutation. (C) Images of spleens from WT and mutant mice.
**Figure S3:** Chromatin features at representative immune gene loci bound by STAT3 and STAT5B. (A) Binding profiles of phosphorylated STAT3 in CD8^+^ T cells stimulated with IL‐6 or IL‐21, and of STAT5B, H3K27ac and RNA polymerase II (Pol II) in total T cells stimulated with IL‐2/IL‐7 from STAT5BY665F mutant mice, at loci co regulated by STAT3 and STAT5B containing canonical GAS motifs. (B) Genomic regions preferentially bound by STAT3, also containing GAS motifs, demonstrating selective regulatory activity distinct from STAT5B.
**Table S1:** DEGs in Stat3‐deficient T cells reconstituted with STAT3 variants, categorised into eight transcriptional clusters. Shown are log_2_ (fold change), adjusted *p*‐values and results of GO enrichment analysis.
**Table S2:** A list of significantly regulated genes in STAT3D661H compared to WT mice, based on normalised read counts from spleen tissue. The list includes log_2_ (fold change), *p*‐values, adjusted *p*‐values and results from GO enrichment analysis. Additionally, a comparison is provided between significantly regulated genes in STAT3D661H and STAT5BY665F.
**Table S3:** STAT3‐ and STAT5B‐binding peaks at GAS motifs identified by ChIP‐seq, including unique and overlapping peaks and their associated potential target genes.
**Table S4:** Comparison between a prior study and the current study using Stat3 mutant mice that model human disease‐associated mutations.
**Table S5:** sgRNA sequences used for CRISPR/Cas9‐mediated Stat3 targeting. Donor oligonucleotides contained the desired amino acid substitutions (GAT→TAT, GAT→GTC or GAT→CAT).
