# Supplementary material for: STAT3 SH2 Domain Aspartic Acid 661 Mutations Activate Immune Gene Programs
Source: J Cell Mol Med. 2026 Jan 13;30(1):e71015. doi: 10.1111/jcmm.71015 (PMC12796846; doi:10.1111/jcmm.71015)
Supplement: Supplementary file 1 — Figure S1: Cross‐species conservation of STAT3 amino acid sequences, with a focus on the region surrounding D661. Figure S2: STAT3 D661 mutations in the mouse genome. (A) Sanger sequencing chromatograms showing wild‐type (WT) and the introduction of SNPs resulting in the two missense mutations, STAT3D661V (D661V) and STAT3D661H (D661H) mutants. The red shade indicates the altered codons converting D661 to D661V and D661H, respectively. (B) Images of founder mice carrying STAT3D661H mutation. (C) Images of spleens from WT and mutant mice. Figure S3: Chromatin features at representative immune gene loci bound by STAT3 and STAT5B. (A) Binding profiles of phosphorylated STAT3 in CD8+ T cells stimulated with IL‐6 or IL‐21, and of STAT5B, H3K27ac and RNA polymerase II (Pol II) in total T cells stimulated with IL‐2/IL‐7 from STAT5BY665F mutant mice, at loci co regulated by STAT3 and STAT5B containing canonical GAS motifs. (B) Genomic regions preferentially bound by STAT3, also containing GAS motifs, demonstrating selective regulatory activity distinct from STAT5B. Table S1: DEGs in Stat3‐deficient T cells reconstituted with STAT3 variants, categorised into eight transcriptional clusters. Shown are log2 (fold change), adjusted p‐values and results of GO enrichment analysis. Table S2: A list of significantly regulated genes in STAT3D661H compared to WT mice, based on normalised read counts from spleen tissue. The list includes log2 (fold change), p‐values, adjusted p‐values and results from GO enrichment analysis. Additionally, a comparison is provided between significantly regulated genes in STAT3D661H and STAT5BY665F. Table S3: STAT3‐ and STAT5B‐binding peaks at GAS motifs identified by ChIP‐seq, including unique and overlapping peaks and their associated potential target genes. Table S4: Comparison between a prior study and the current study using Stat3 mutant mice that model human disease‐associated mutations. Table S5: sgRNA sequences used for CRISPR/Cas9‐mediated Stat3 [file JCMM-30-e71015-s001.zip › jcmm71015-sup-0001-FigureS1-S3-TableS5.pdf]

Supplementary information

STAT3 SH2 domain aspartic acid 661 mutations activate immune gene programs

Hye Kyung Lee[1], Gyuhyeok Cho[2], Jichun Chen[3], Aaron B. Schultz[4, 5], Sung-Gwon Lee[1], Chengyu Liu[6], Priscilla A. Furth[1], Neal S. Young[3], Jungwook Kim[2], Alejandro Villarino[4, 5] and Lothar Hennighausen[1, *]

Supplementary Figures

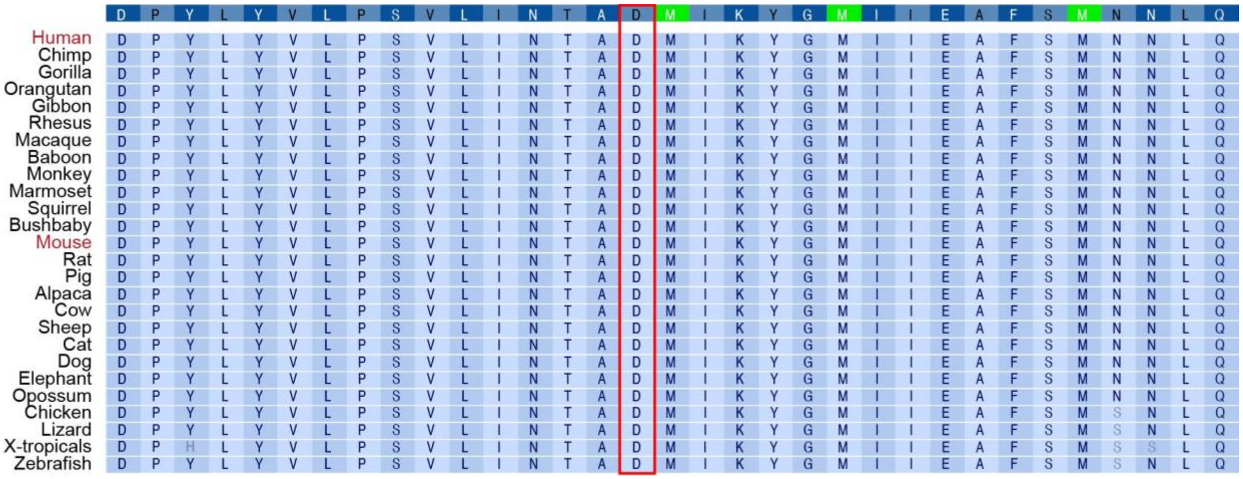

Supplementary Figure 1. Cross-species conservation of STAT3 amino acid sequences, with a focus on the region surrounding D661.

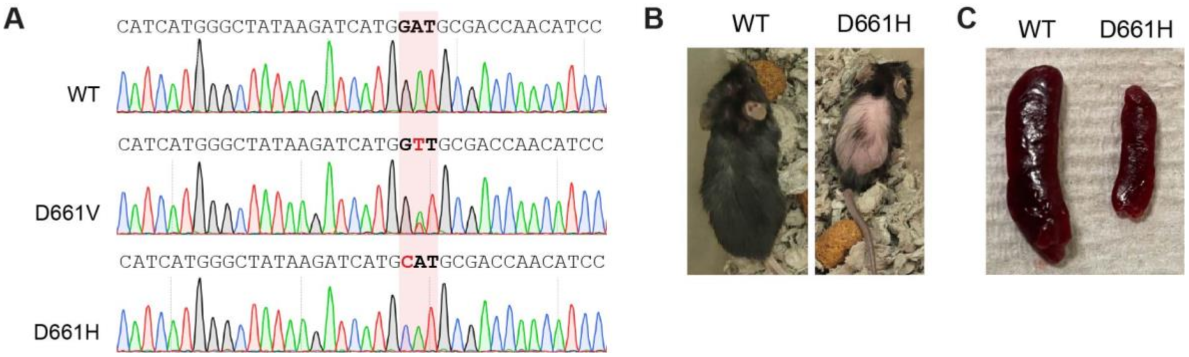

Supplementary Figure 2. STAT3 D661 mutations in the mouse genome. (A) Sanger sequencing chromatograms showing wild-type (WT) and the introduction of SNPs resulting in the two missense mutations, STAT3^{D661V} (D661V) and STAT3^{D661H} (D661H) mutants. The red shade indicates the altered codons converting D661 to D661V and D661H, respectively. **(B)** Images of founder mice carrying STAT3^{D661}H mutation. **(C)** Images of spleens from WT and mutant mice.

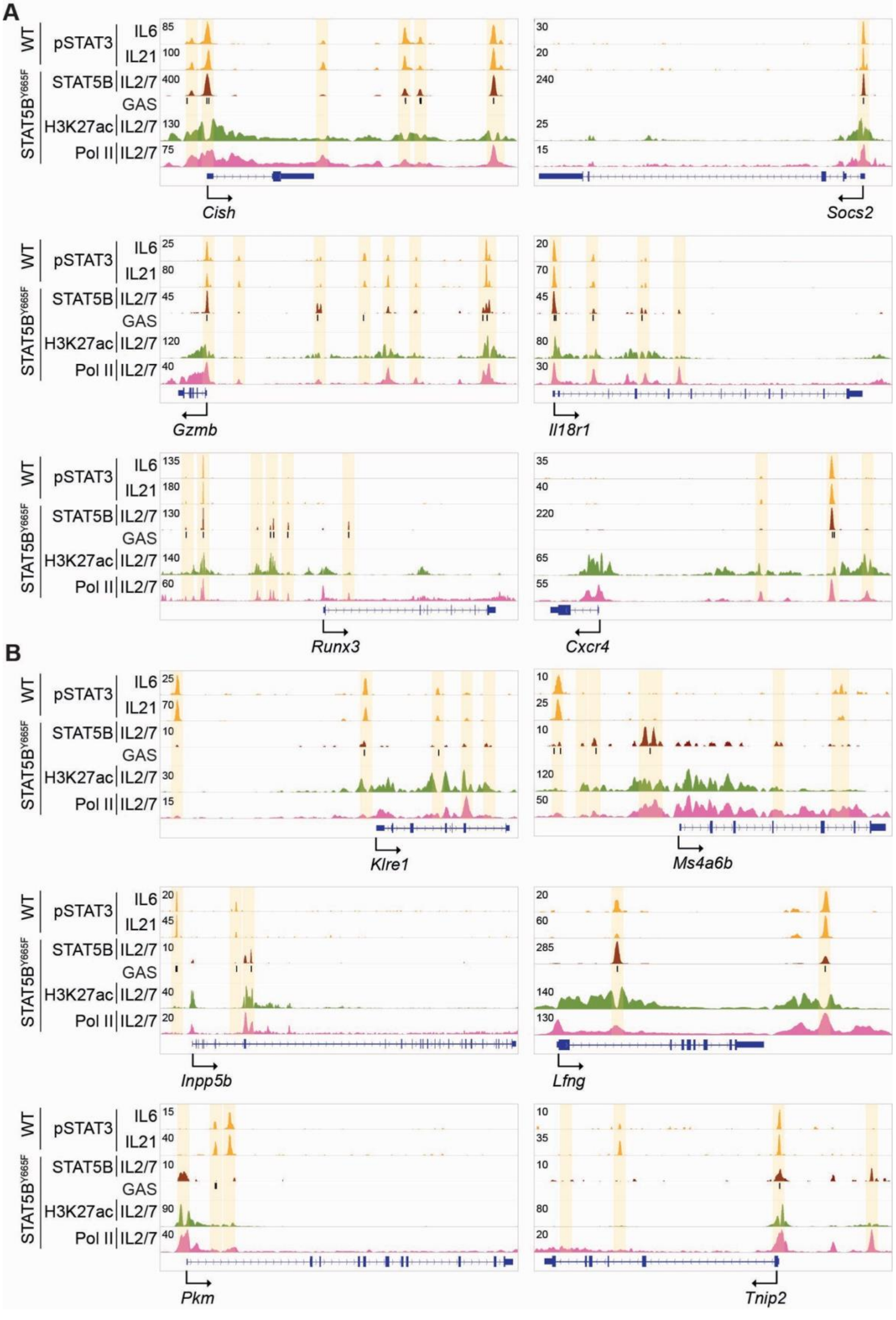
A
B
WT
pSTAT3
IL6
IL21
STAT5B^{Y665F}
STAT5B
IL2/7
GAS
H3K27ac
Pol II
Cish
Socs2
Gzmb
Il18r1
Runx3
Cxcr4
Klre1
Ms4a6b
Inpp5b
Lfng
Pkm
Tnip2

Supplementary Figure 3. Chromatin features at representative immune gene loci bound by STAT3 and STAT5B. (A) Binding profiles of phosphorylated STAT3 in $CD8^+$ T cells stimulated with IL-6 or IL-21, and of STAT5B, H3K27ac, and RNA polymerase II (Pol II) in total T cells stimulated with IL-2/IL-7 from $STAT5B^{Y665F}$ mutant mice, at loci co-regulated by STAT3 and STAT5B containing canonical GAS motifs. **(B)** Genomic regions preferentially bound by STAT3, also containing GAS motifs, demonstrating selective regulatory activity distinct from STAT5B.

Supplementary Tables

Supplementary Table 1. DEGs in Stat3-deficient T cells reconstituted with STAT3 variants, categorized into eight transcriptional clusters. Shown are log2(fold change), adjusted p-values, and results of GO enrichment analysis.

Supplementary Table 2. A list of significantly regulated genes in $STAT3^{D661H}$ compared to WT mice, based on normalized read counts from spleen tissue. The list includes $\log_2$(fold change), p-values, adjusted p-values, and results from GO enrichment analysis. Additionally, a comparison is provided between significantly regulated genes in $STAT3^{D661H}$ and $STAT5B^{Y665F}$.

Supplementary Table 3. STAT3- and STAT5B-binding peaks at GAS motifs identified by ChIP-seq, including unique and overlapping peaks, and their associated potential target genes.

Supplementary Table 4. Comparison between a prior study and the current study using Stat3 mutant mice that model human disease-associated mutations.

Supplementary Table 5. sgRNA sequences used for CRISPR/Cas9-mediated Stat3 targeting. Donor oligonucleotides contained the desired amino acid substitutions (GAT→TAT, GAT→GTC, or GAT→CAT).

Target site	sgRNA sequences
$STAT3^{D661Y}$	5'-CATCATGGGCTATAAGATCA-3'<br><br>Donor Oligos<br>CCAAGCAGCAGCTGAACAACATGTCATTTGCTGAAATCATCATGGGCTATAAGATCATGTATGCG<br>ACCAACATCCTGGTGTCTCCACTTGTCTACCTCTACCCCGACATTCCCAAGGAGGAGGCATTTGG
$STAT3^{D661V}$	5'-CATGGATGCGACCAACATCC-3'<br><br>Donor Oligos<br>CCAAGCAGCAGCTGAACAACATGTCATTTGCTGAAATCATCATGGGCTATAAGATCATGGTCGCG<br>ACC AACATCCTCGTGTCTCCACTTGTCTACCTCTACCCCGACATTCCCAAGGAGGAGGCATTTGG
$STAT3^{D661H}$	5'-CATCATGGGCTATAAGATCA-3'<br><br>Donor Oligos<br>CCAAGCAGCAGCTGAACAACATGTCATTTGCTGAAATCATCATGGGCTATAAGATCATGCATGCG<br>ACCAACATCCTGGTGTCTCCACTTGTCTACCTCTACCCCGACATTCCCAAGGAGGAGGCATTTGG
